# Combined 595-nm pulsed dye laser and picosecond laser toning for the treatment of extensive erythema ab igne: A case report

**DOI:** 10.1016/j.jdcr.2026.08.024

**Published:** 2026-08-23

**Authors:** Lydia C. Hesseltine, Ronald J. Sulewski

**Affiliations:** a University of Nebraska Medical Center, College of Medicine, Omaha, Nebraska; b Department of Dermatology, University of Nebraska Medical Center, Omaha, Nebraska

**Keywords:** erythema ab igne, hyperpigmentation, laser toning, picosecond laser, pulsed dye laser

## Introduction

Erythema ab igne (EAI) is a reticulated dermatosis resulting from repeated exposure to moderate heat or infrared radiation insufficient to produce thermal burns.^1^ Historically associated with fireplaces and stoves, contemporary cases arise from chronic use of heating pads, laptop computers, space heaters, and automotive seat warmers.^1^^,^^2^

Persistent thermal exposure induces vascular dilation, epidermal atrophy, hemosiderin deposition, and melanin incontinence, producing characteristic reticulated erythematous-to-brown discoloration.^1^^,^^3^ Although heat cessation may lead to gradual improvement, persistent hyperpigmentation and telangiectatic erythema can remain for months to years and cause cosmetic distress.^3^ Therapeutic options remain poorly defined. Topical agents (tretinoin, hydroquinone, 5-fluorouracil) have shown variable efficacy.^2^ Reports have demonstrated improvement with pigment-targeting laser therapies, including low-fluence 1064-nm Q-switched neodymium-doped yttrium aluminum garnet (Nd:YAG) laser combined with topical hydroquinone, and 755-nm picosecond alexandrite laser.^4^^,^^5^ A systematic review of picosecond lasers in dermatology demonstrated favorable safety profiles with lower risk of post-inflammatory hyperpigmentation compared to nanosecond Q-switched lasers across multiple pigmentary indications.^6^ However, literature regarding vascular laser treatment in EAI remains extremely limited.

EAI shares mixed vascular and pigmentary pathology with other conditions. Poikiloderma of Civatte and melasma are characterized by a combination of pigment and vascular abnormalities. Pulsed dye laser (PDL) and intense pulsed light treatments are well established in improving poikiloderma.^7^ Targeting the vascular component of melasma is a newer approach with demonstrated benefit and acceptable safety.^8^

Given this overlap, combined vascular and pigment-directed laser therapy may provide synergistic benefit in EAI. We report a case of extensive EAI involving the back successfully treated with sequential 595-nm PDL and 1064-nm picosecond laser toning.

## Case report

A 38-year-old woman with Fitzpatrick skin type II presented with asymptomatic reticulated hyperpigmentation involving the entire back. Her medical history included chronic low back pain secondary to lumbar spondylosis, managed with opioid analgesics and nightly application of an electric heating pad directly to the back for 8–10 hours nightly over 1 year.

After six months of heat exposure, she developed diffuse erythema across the back that progressed to reticulated hyperpigmented patches, which intensified with continued heating pad use. Prior topical retinol treatment was ineffective.

Physical examination demonstrated diffuse background erythema with extensive, well-demarcated reticulated hyperpigmented patches involving the back (Fig 1). Dermoscopic examination revealed gray granules, background erythema, and telangiectasias. Given the characteristic morphology and exposure history, the diagnosis was clinical and biopsy was deferred.

**Fig 1:**
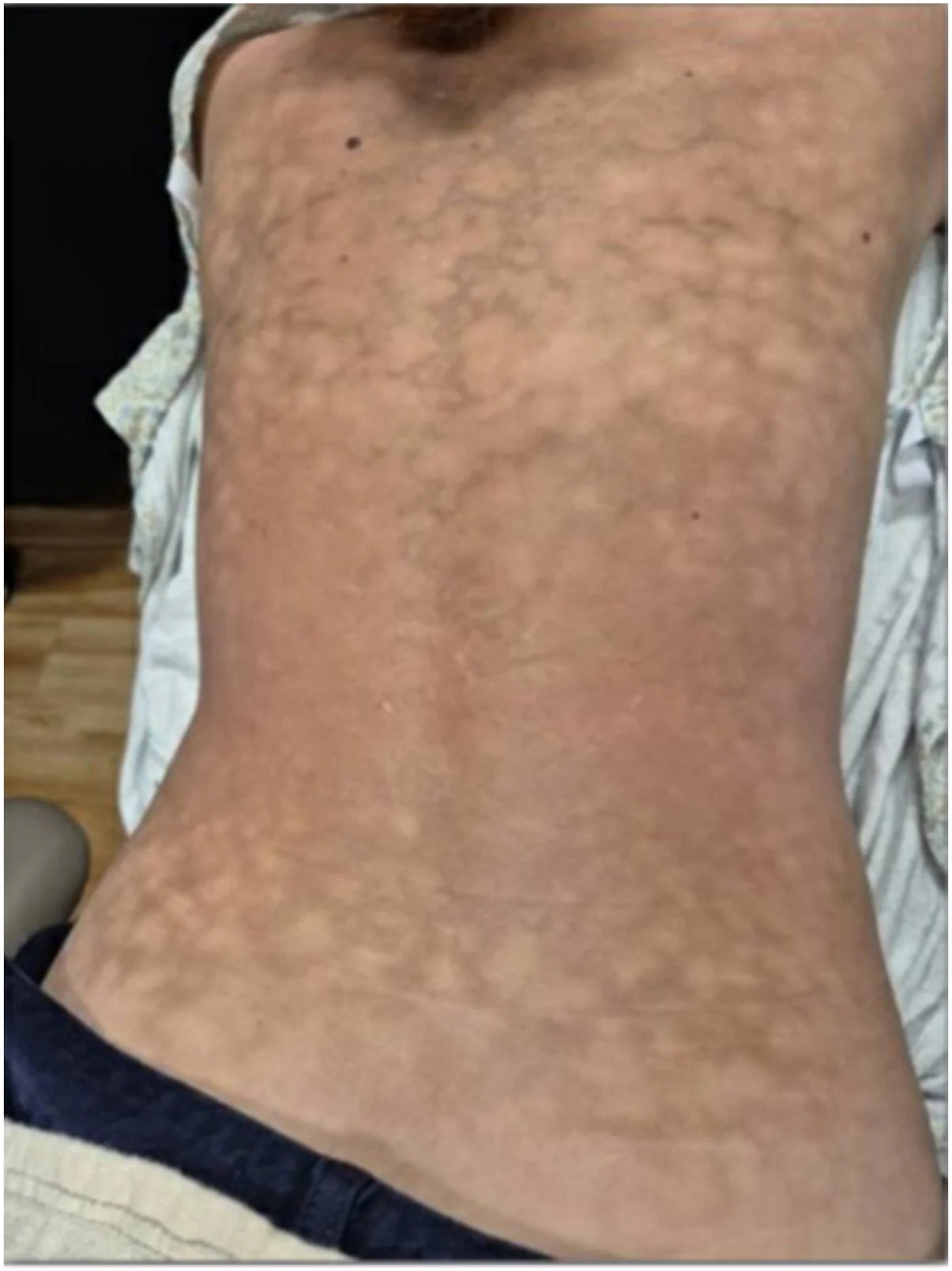
Extensive reticulated hyperpigmentation and background erythema involving the back prior to treatment.

Owing to the extensive body surface area involved and prominent erythematous component, treatment with a 595-nm PDL was initiated (Vbeam Prima, Candela Wayland, MA). The patient had discontinued heating pad use several months prior to the first treatment. The patient underwent 3 sessions of PDL therapy at 4- to 6-week intervals using the following parameters: fluence 6.25 to 6.75 J/cm^2^, spot size 15 mm, pulse duration 10 ms. Following improvement in erythema, residual reticulated hyperpigmentation was subsequently treated with 3 sessions of picosecond laser toning at 6-week intervals (Picoway, Candela Resolve 1064 nm handpiece) fluence 1.5 to 1.9 J/cm^2^, 6 × 6 mm, repetition rate 10 Hz, 4 passes per session. Total treatment duration was approximately ten months.

Treatment was well tolerated. Following PDL therapy, erythema and telangiectasias improved clinically (Fig 2). Residual reticulated hyperpigmentation progressively lightened after picosecond laser toning (Fig 3).

**Fig 2:**
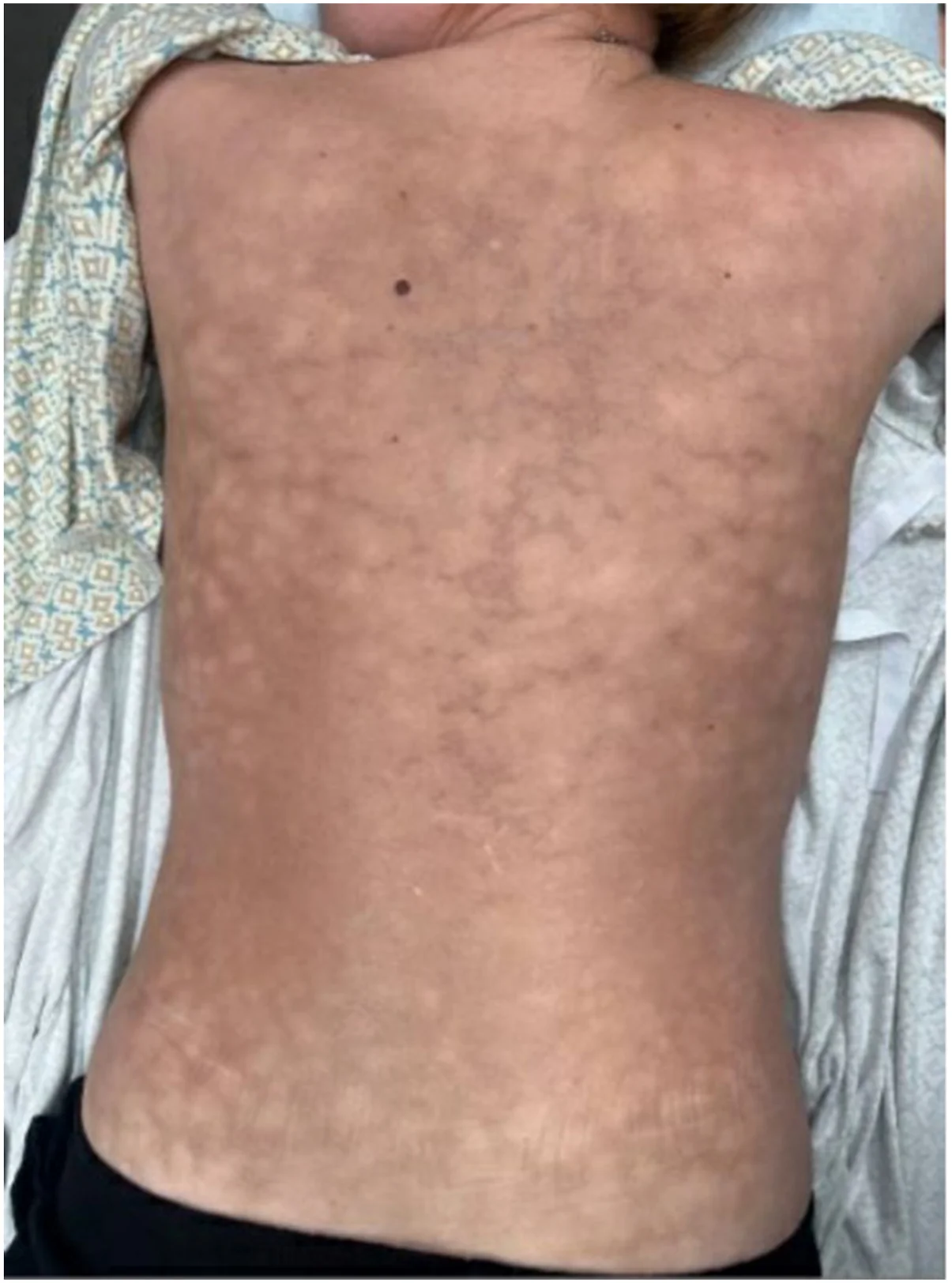
Improvement in erythema and telangiectatic changes following three sessions of 595-nm pulsed dye laser therapy.

**Fig 3:**
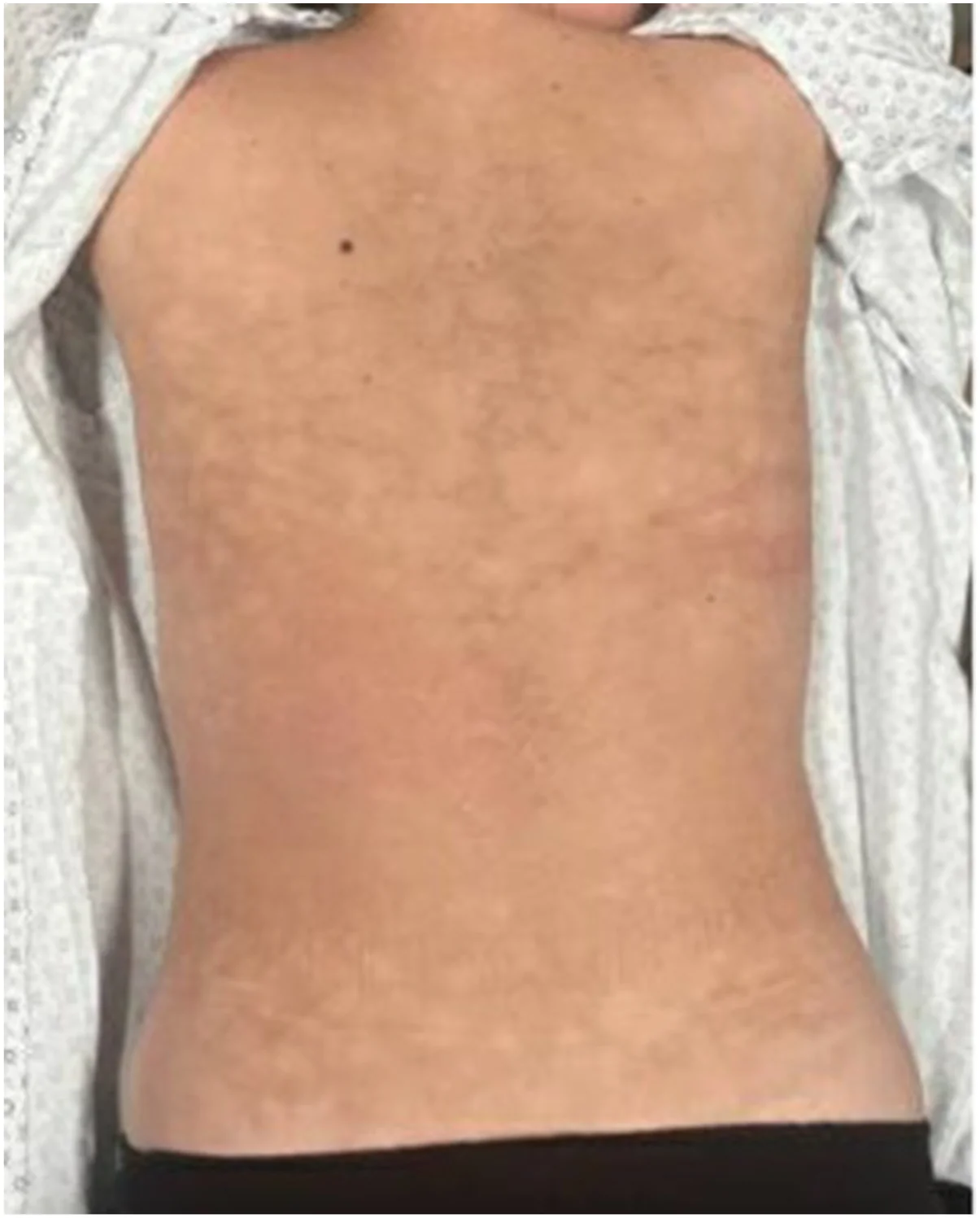
Further improvement in reticulated hyperpigmentation following three sessions of 1064-nm picosecond laser toning.

No ulceration, scarring, dyspigmentation, or paradoxical worsening was observed during 12 months of follow-up. The patient reported satisfaction with the cosmetic outcome.

## Discussion

EAI is a chronic, thermally-induced dermatosis characterized by reticulated erythema and hyperpigmentation resulting from prolonged infrared exposure.^1^ Histopathologic findings may include epidermal atrophy, basal keratinocyte atypia, melanin and hemosiderin deposition, and superficial vascular dilation.^1^^,^^3^ Chronic lesions may persist despite heat cessation and rarely undergo malignant transformation.^2^

Management of EAI centers on discontinuation of heat exposure. In addition to heat cessation, routine sun protection should be encouraged to reduce cumulative photodamage and support overall skin health. However, residual dyspigmentation may remain cosmetically distressing and refractory to conservative measures. Published evidence supporting laser therapy in EAI remains sparse and largely limited to isolated case reports. Previous reports have described successful treatment of EAI with Q-switched Nd:YAG and picosecond alexandrite lasers, supporting a role for pigment-targeted photothermolysis.^4^^,^^5^

To our knowledge, reports evaluating PDL therapy in EAI are extremely limited. Nevertheless, the vascular component of EAI provides a strong rationale for PDL treatment. PDL selectively targets oxyhemoglobin and is well established for vascular dermatoses including telangiectasias, poikiloderma, and post-inflammatory erythema.^7^^,^^9^ This approach parallels emerging melasma treatment strategies in which targeting the vascular component renders pigmentary improvement more attainable.^8^

In this case, 595-nm PDL improved erythema and vascular changes, while subsequent picosecond laser toning addressed residual reticulated hyperpigmentation. A systematic review supports picosecond 1064-nm toning, demonstrating efficacy across pigmentary indications with lower risk of post-inflammatory hyperpigmentation than nanosecond Q-switched lasers.^6^ A split-face randomized controlled trial comparing picosecond and nanosecond Nd:YAG 1064-nm lasers for melasma found equivalent efficacy but less pain and post-procedure erythema with picosecond technology, further supporting its use for pigmentary conditions.^10^ Although melasma and EAI are distinct, these data support the safety and pigment-targeting efficacy of picosecond 1064-nm toning.

This case suggests that sequential vascular and pigment-directed laser therapy may represent a useful strategy for extensive or cosmetically significant EAI, drawing on established treatment principles from poikoloderma of Civatte and melasma. Concurrent PDL and picosecond laser therapy, rather than a stepwise approach, may also be effective.

There is currently no direct evidence quantifying a reduction in risk of squamous cell carcinoma in EAI following laser treatment. However, Q-switched 1064-nm Nd:YAG laser has been shown to clear epithelial atypia, which is the histologic precursor to malignant transformation, potentially leading to reduction in subsequent risk of squamous cell carcinoma. This rationale is largely theoretical, and ongoing surveillance of longstanding EAI lesions is recommended.^2^

Limitations include single-patient design, absence of histopathologic confirmation, and lack of objective outcome measures. It should be noted that improvement attributable to heat cessation, which is the mainstay of treatment, cannot be excluded. However, the temporal relationship between heat cessation, laser treatment, and clinical improvement suggests therapeutic benefit. Prospective studies with objective outcome measures are needed to establish standardized laser parameters and long-term outcomes in EAI.

### Declaration of generative AI and AI-assisted technologies in the writing process

None.

## Conflicts of interest

None disclosed.
